# 12-Hydroxyeicosapentaenoic acid inhibits foam cell formation and ameliorates high-fat diet-induced pathology of atherosclerosis in mice

**DOI:** 10.1038/s41598-021-89707-1

**Published:** 2021-05-17

**Authors:** Takahiro Nagatake, Yuki Shibata, Sakiko Morimoto, Eri Node, Kento Sawane, So-ichiro Hirata, Jun Adachi, Yuichi Abe, Junko Isoyama, Azusa Saika, Koji Hosomi, Takeshi Tomonaga, Jun Kunisawa

**Affiliations:** 1 Laboratory of Vaccine Materials, Center for Vaccine and Adjuvant Research and Laboratory of Gut Environmental System, National Institutes of Biomedical Innovation, Health and Nutrition (NIBIOHN), 7-6-8 Asagi Saito, Ibaraki, Osaka 567-0085 Japan; 2 Graduate School of Pharmaceutical Sciences, Osaka University, 1-1 Yamadaoka, Suita, Osaka 565-0871 Japan; 3 Department of Microbiology and Immunology, Kobe University Graduate School of Medicine, 7-5-1 Kusunoki-cho, Chuo-ku, Kobe, Hyogo 650-0017 Japan; 4 Laboratory of Proteome Research and Laboratory of Proteomics for Drug Discovery, NIBIOHN, 7-6-8 Asagi Saito, Ibaraki, Osaka 567-0085 Japan; 5 Division of Molecular Diagnosis, Aichi Cancer Center Research Institute, 1-1 Kanokoden, Chikusa-ku, Nagoya, 464-8681 Japan; 6 International Research and Development Center for Mucosal Vaccines, The Institute of Medical Science, The University of Tokyo, 4-6-1 Shirokanedai, Minato-ku, Tokyo, 108-8639 Japan; 7 Graduate School of Medicine, Graduate School of Dentistry, Department of Science, Osaka University, 1-1 Yamadaoka, Suita, Osaka 565-0871 Japan

**Keywords:** Inflammation, Cardiovascular diseases, Lipidomics

## Abstract

Atherosclerosis is a chronic inflammatory disease associated with macrophage aggregate and transformation into foam cells. In this study, we sought to investigate the impact of dietary intake of ω3 fatty acid on the development of atherosclerosis, and demonstrate the mechanism of action by identifying anti-inflammatory lipid metabolite. Mice were exposed to a high-fat diet (HFD) supplemented with either conventional soybean oil or α-linolenic acid-rich linseed oil. We found that as mice became obese they also showed increased pulsatility and resistive indexes in the common carotid artery. In sharp contrast, the addition of linseed oil to the HFD improved pulsatility and resistive indexes without affecting weight gain. Histological analysis revealed that dietary linseed oil inhibited foam cell formation in the aortic valve. Lipidomic analysis demonstrated a particularly marked increase in the eicosapentaenoic acid-derived metabolite 12-hydroxyeicosapentaenoic acid (12-HEPE) in the serum from mice fed with linseed oil. When we gave 12-HEPE to mice with HFD, the pulsatility and resistive indexes was improved. Indeed, 12-HEPE inhibited the foamy transformation of macrophages in a peroxisome proliferator-activated receptor (PPAR)γ-dependent manner. These results demonstrate that the 12-HEPE-PPARγ axis ameliorates the pathogenesis of atherosclerosis by inhibiting foam cell formation.

## Introduction

Omega-3 and ω6 fatty acids are polyunsaturated fatty acids in which the first double bond is located on the third and sixth carbon atom, respectively, from the methyl terminal of the hydrocarbon chain. They are essential fatty acids that cannot be synthesized in vivo by humans or other mammals, and must therefore be ingested in food. The quality of dietary oil intake is thus directly related to the amounts of ω3 and ω6 fatty acids in the body. A range of vegetable cooking oils are commercially available, and the soybean oil used in the regular chow for laboratory mice contains approximately 5% α-linolenic acid, which is an ω3 fatty acid. However, the α-linolenic acid content of linseed oil is approximately 60%, which is at least 10 times that of soybean oil.

In a previous study, we found that the use of chow containing linseed oil instead of soybean oil suppressed food allergy, and identified 17,18-epoxyeicosatetraenoic acid (17,18-EpETE), a metabolite of eicosapentaenoic acid (EPA), as the active molecule that possesses anti-allergic activity^1^. Further analyses using a contact dermatitis model revealed that 17,18-EpETE blocks neutrophil migration via the G-protein-coupled receptor GPR40, and exerts an inhibitory effect on dermatitis^2,3^. We have also shown that linseed oil ingestion improves allergic rhinitis, and identified the EPA metabolite 15-hydroxyeicosapentaenoic acid as the active metabolite^4^. Furthermore, we have found the pathway through which the anti-allergic effect of ω3 fatty acid ingestion is transmitted to infants via breast milk, and identified the active ingredient in this case as the n-3 docosapentaenoic acid (n-3 DPA) metabolite 14-hydroxy-DPA^5^. Also, recent studies by other group have reported a number of other ω3 fatty acid metabolites, such as resolvins, protectins, and maresins, which have been found to possess potent anti-inflammatory and inflammation-resolving effects, and these are attracting great attention as new mechanisms of action of ω3 fatty acids^6,7^. On the basis of these findings, it is becoming generally accepted that when ω3 fatty acids ingested in the diet are converted in vivo to active molecules, they show effectiveness against allergies and inflammatory diseases.

Atherosclerosis is a chronic inflammatory disease, which is characterized by the presence of plaques, which form when macrophages aggregate around the vascular wall and are transformed into foam cells. Obesity is regarded as one of the factors contributing to the disease^8–10^. Mechanistically, obesity causes increasement of vascular permeability, which enables LDL to leak from the blood into the vascular sub-endothelium, where it is oxidized by active oxygen species generated in endothelial cells to form oxidized LDL (Ox-LDL)^11^. The binding of Ox-LDL to the Ox-LDL receptor Lox-1 expressed by endothelial cells activates these cells, inducing not only the production of inflammatory cytokines such as IL-1 and tumor necrosis factor (TNF)-α, as well as that of chemokine CCL2, but also the expression of the cell adhesion molecules vascular cell adhesion molecule (VCAM)-1 and E-/P-selectin^12,13^. Because monocytes carried in the blood express the E-/P-selectin ligand PSGL-1, they roll along the surface of vascular endothelium, which expresses E-/P-selectin^12,13^. When the integrin β1 in monocytes is activated by CCL2-mediated signals, this causes it to interact strongly with VCAM-1, resulting in strong adhesion^14–16^. The expansion of the intercellular space resulting from endothelial cell activation also enables monocytes to migrate into the sub-endothelium, where macrophage colony-stimulating factor produced by endothelial cells causes them to differentiate into macrophages^17,18^. The macrophages that have differentiated in the sub-endothelium then phagocytose Ox-LDL via the Ox-LDL receptors CD36 and SR-A, and when Ox-LDL breaks down within the cells it accumulates intracellularly as cholesterol esters^17,18^. Repeated phagocytosis causes the macrophages to become hypertrophic, transforming them into foamy macrophages; this is defined as fatty streak^17–19^. Fatty streak proceeds to the formation of fibrous plaque and advanced plaque, which shows infiltration of smooth muscle cells and accumulation of extracellular matrix, and release of matrix metalloproteinases, respectively^19^. Finally, degradation of the extracellular matrix by metalloproteinases increases plaque vulnerability and their breakdown leads to the formation of blood clots that occlude the vessel and obstruct blood flow, causing acute myocardial infarction, cerebral infarction, and other forms of cardiovascular disease^19^. Atherosclerosis is thus an underlying condition for cardiovascular disease, and depends on the chronic inflammatory reaction caused by the interaction between macrophages and endothelial cells.

As a result of previous epidemiological studies that have found that EPA intake inhibits cardiovascular disease^20–22^, EPA formulations are now widely used as atherosclerosis medications. In light of the functions of metabolites newly identified in recent ω3 fatty acid research, it is highly likely that EPA exerts its physiological action as a result of its metabolism in vivo. In this study, we verified the effectiveness of ω3 fatty acid ingestion against the pathogenesis of atherosclerosis, and showed that the EPA metabolite 12-hydroxyeicosapentaenoic acid (12-HEPE) suppresses the development of atherosclerosis as an active metabolite that inhibits the transformation of macrophages into foam cells in a peroxisome proliferator-activated receptor (PPAR)γ-dependent manner.

## Results

### Dietary linseed oil inhibited the development of HFD-induced fatty streak without affecting body weight increase

**Figure 1:**
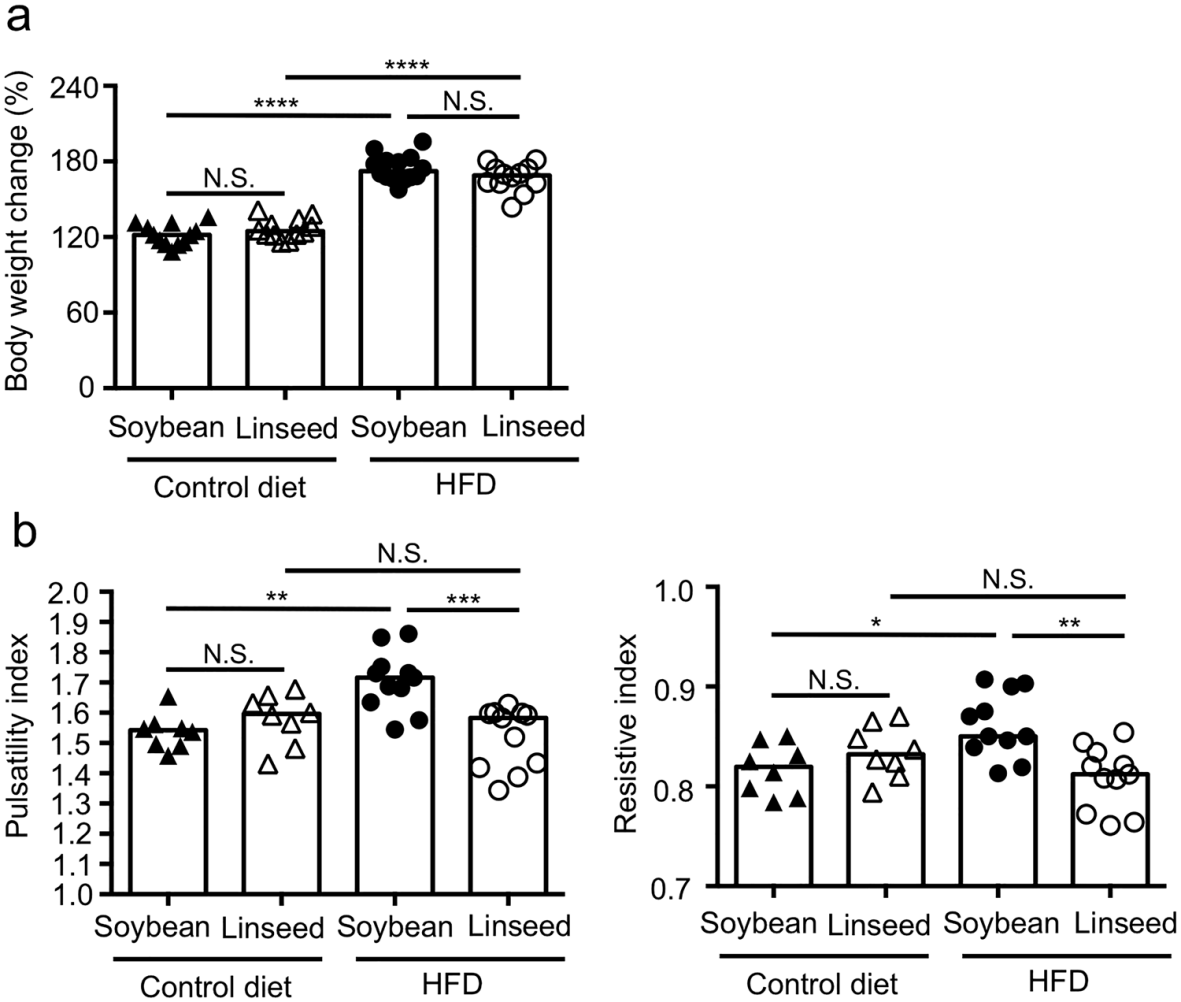
HFD-induced atherosclerosis was ameliorated by dietary linseed oil. Mice were fed with either control diet with soybean oil, control diet with linseed oil, HFD with soybean oil or HFD with linseed oil for 2 months, and their body weight changes (**a**) and pulsatility and resistive indexes in common carotid artery (**b**) were examined. Data are combined from 3 independent experiments. Each point represents data from individual mice; (**a**) n = 12–14/group, (**b**) n = 8–11/group. Statistical significance of differences was evaluated by means of 1-way ANOVA; **p < 0.01; ***p < 0.001; ****p < 0.0001; *N.S*. not significant.

We first sought to examine the impact of dietary intake of ω3 fatty acid in the development of obesity and atherosclerosis. To address this issue, we prepared 4 different types of diets; control diets that contain conventional amount 4% (wt/wt) of either soybean oil or linseed oil, and HFDs that contain lard and beef tallow which were supplemented with 10% (wt/wt) of either soybean oil or linseed oil, and maintained mice for 2 months on either the diet, and measured the body weight and pulsatility index and resistive index as indicators of the development of atherosclerosis^23–25^. We found that HFD-fed mice showed increased body weight when compared with control diet-fed mice, however, there were no differences between two types of dietary oils, suggesting that dietary ω3 fatty acids have no effect on the development of obesity (Fig. 1a). In spite of little effects on obesity, analysis of pulsatility index and resistive index in the common carotid artery indicated that dietary linseed oil inhibited the development of atherosclerosis (Fig. 1b). Similar results were obtained from mice fed with HFD for 4 months and 6 months (Supplementary Fig. S1).

**Figure 2:**
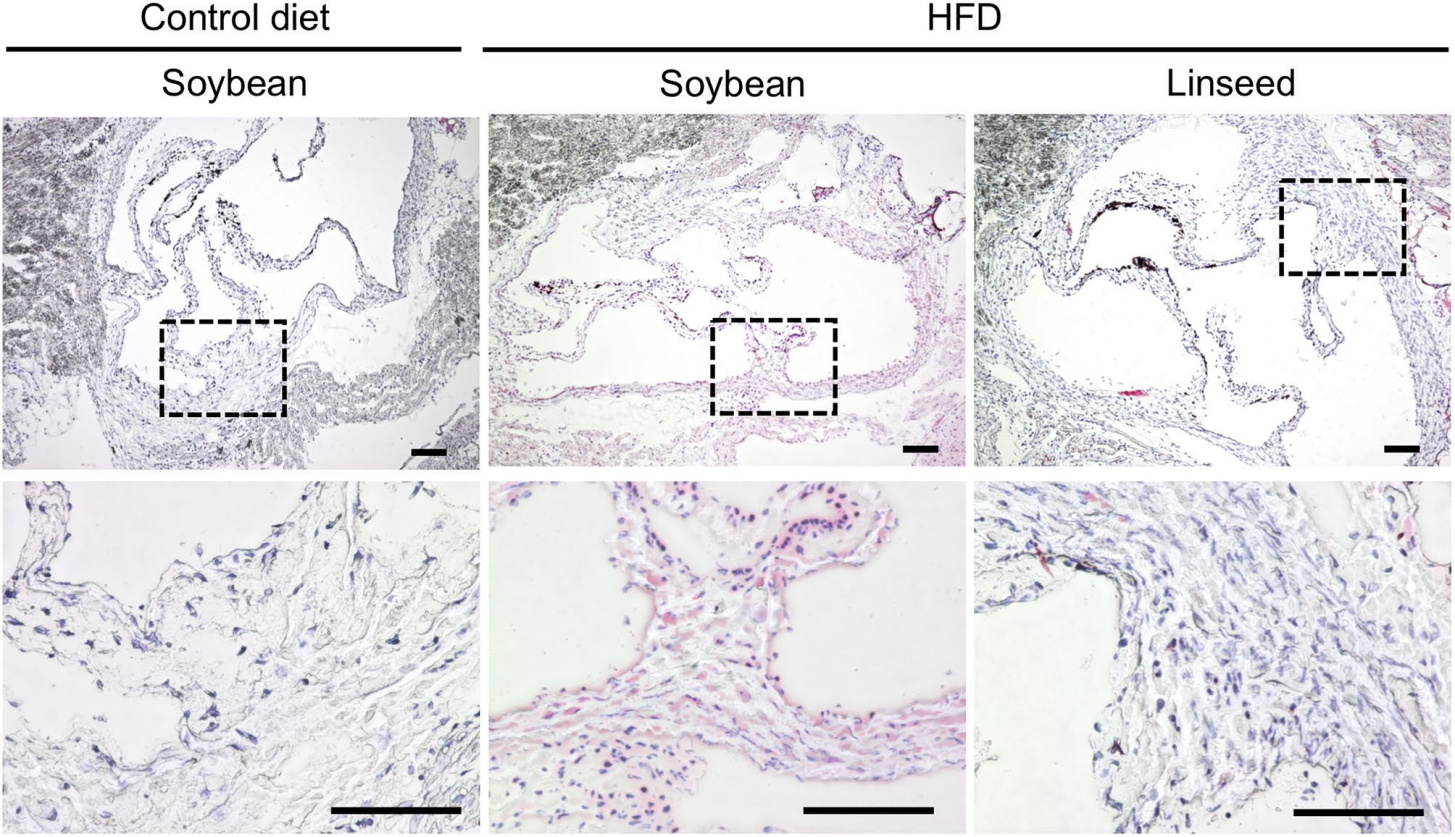
HFD-induced tissue deposition of lipid was inhibited by dietary linseed oil. Mice were fed with either control diet with soybean oil, HFD with soybean oil or HFD with linseed oil for 10 months, and histological sections of aortic valve were examined by staining with oil red O solution. Upper row shows pictures with low magnification. Lower tier shows pictures with high magnification of indicated square areas. Data are representative of 2 independent experiments (n = 5/group/experiment). Scale bars 100 μm.

**Figure 3:**
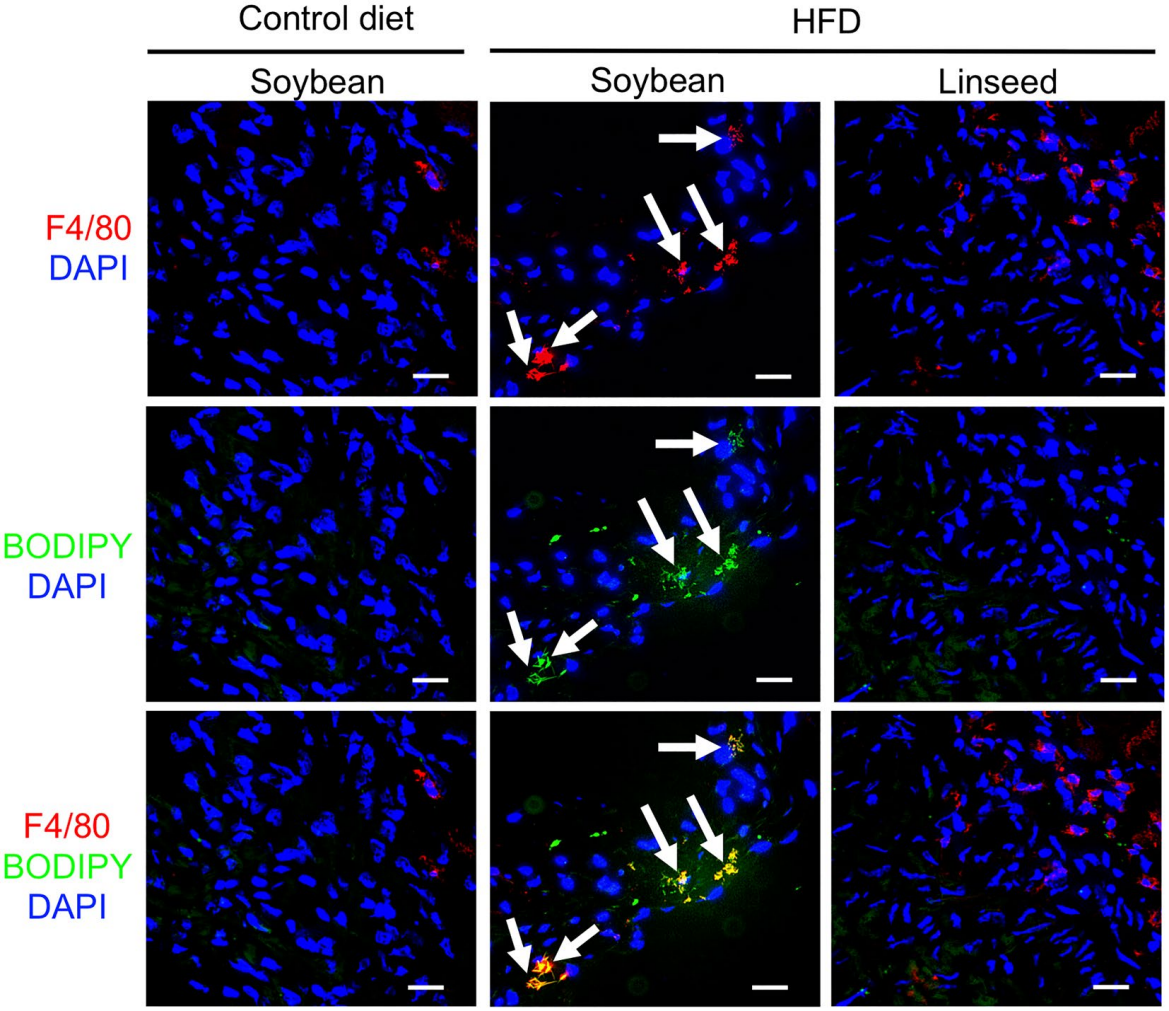
HFD-induced foam cell formation was inhibited by dietary linseed oil. Mice were fed with either control diet with soybean oil, HFD with soybean oil or HFD with linseed oil for 10 months, and histological sections of aortic valve were examined by staining with indicated antibody and reagents. Data are representative of 2 independent experiments (n = 5/group/experiment). Arrows indicate the development of foam cells. Scale bars 20 μm.

Given that pulsatility index was improved by dietary linseed oil, it was reasonable to next examine whether histological analysis supports the findings. To address this issue, we analyzed histological sections of aortic valve by staining with oil red O solution to identify lipid deposition to the tissue. In accordance with the pulsatility index results, mice fed with HFD supplemented with soybean oil showed the presence of lipid deposition in the tissue and those signals were not observed in mice fed with HFD supplemented with linseed oil as well as mice fed with control diet (Fig. 2). However, compared with frequently used atherosclerosis models of *Apoe*^−/−^ mice and *Ldlr*^−/−^ mice^26,27^, current investigation by using wild type mice with HFD led to the oil deposit to a milder extent, and the signals were detected inside the cells, but not detected as plaques. Also, it was found that the level of serum LDL/VLDL and HDL was increased in HFD mice when compared with control diet (Supplementary Fig. S2), but the levels were again not much of *Apoe*^−/−^ mice and *Ldlr*^−/−^ mice^26,27^. Immunohistochemical analysis of the tissue sections revealed that macrophages (F4/80^+^) that bear lipid droplet (BODIPY^+^) appeared in mice fed with HFD supplemented with soybean oil but not linseed oil or control diet (Fig. 3). Flow cytometric analysis found that the numbers of macrophages in the aortic valve were comparable between two HFD groups (Supplementary Fig. S3). These findings suggest that the current animal model using wild-type mice with HFD for a long period develop up to the state of fatty streak, which was inhibited by dietary linseed oil.

### EPA-metabolite 12-HEPE markedly increased in serum by dietary linseed oil

**Figure 4:**
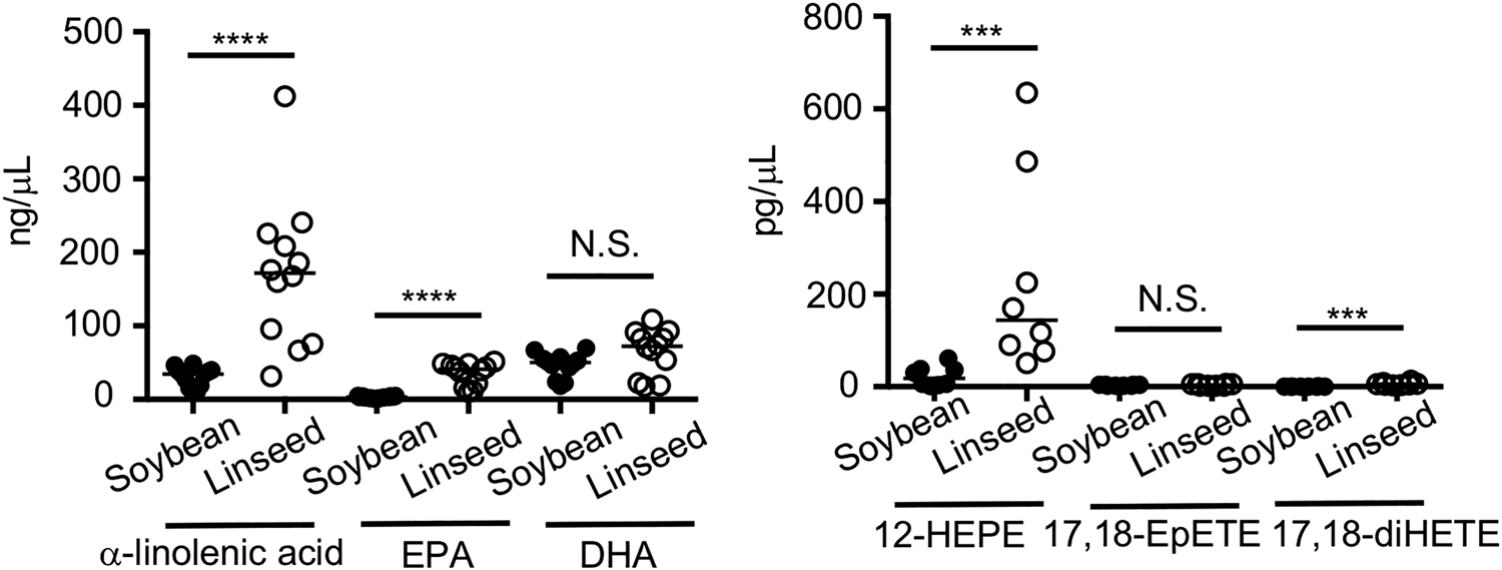
Dietary linseed oil increased the amount of 12-HEPE in serum. Mice were fed with either HFD with soybean oil or HFD with linseed oil for 6 months, and serum was examined by LC–MS/MS for the quantification of fatty acid metabolites. Data are combined from 2 independent experiments. Each point represents data from individual mice; n = 8–12/group. Center values indicate median. Statistical significance of differences was evaluated by means of Mann–Whitney test; ****p < 0.0001; ***p < 0.001; N.S. not significant.

We next performed LC–MS/MS analysis to examine metabolic progression of ω3 fatty acids in serum. In line with fatty acid composition of dietary oils, we found that the amount of α-linolenic acid was increased in serum of mice fed with HFD with linseed oil when compared with HFD with soybean oil (Fig. 4). We also found increased amounts of α-linolenic acid-derived metabolites, EPA and docosahexaenoic acid (DHA), in serum of linseed oil-fed mice (Fig. 4). Several lines of evidence suggest that fatty acid metabolism by cyclooxygenases (COXs), lipoxygenases (LOXs) and cytochrome P450s (CYPs) is a key process to exert their potent bioactivity, which prompted us to next investigate fatty acid-derived mediator profiles. Among mediators examined, we found that the amount of 12-HEPE was markedly increased in serum of mice fed with HFD supplemented with linseed oil when compared with HFD supplemented with soybean oil (Fig. 4). Although the amount of 17,18-diHETE also increased in linseed oil-fed groups, its elevation levels was small when compared with 12-HEPE, suggesting that metabolic progression of ω3 fatty acids is different between serum and intestine^1^.

### 12-HEPE injection improved the pulsatility and resistive indexes in common carotid artery

**Figure 5:**
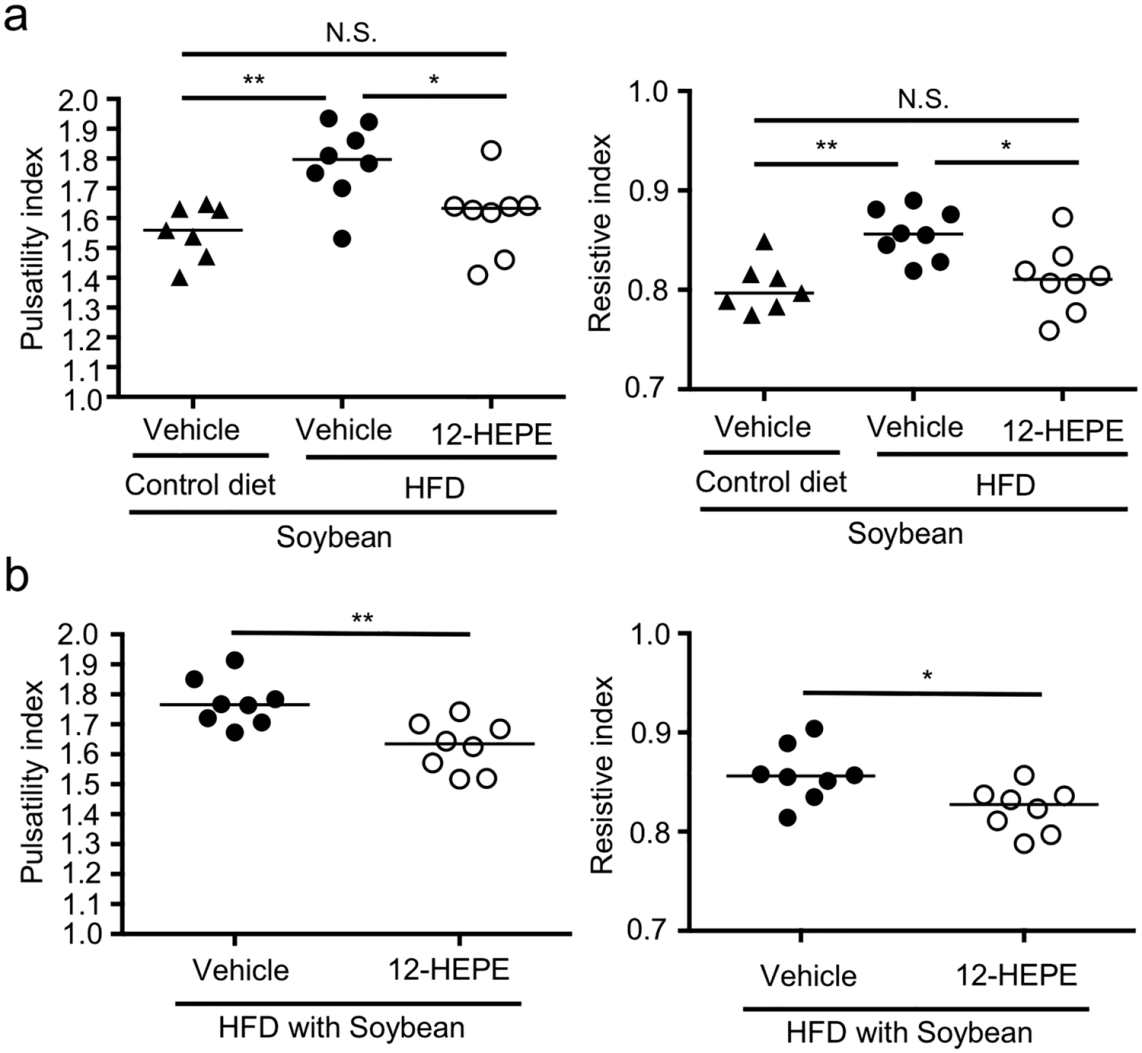
12-HEPE exerted anti-atherosclerosis activity. (**a**) Mice were fed with either control diet with soybean oil or HFD with soybean oil for 2 months. During that time, mice were intraperitoneally injected with either 12-HEPE (100 ng/mouse) or vehicle 3 times/week, and then pulsatility index and resistive index were examined. Data are combined from 2 independent experiments. Each point represents data from individual mice. Statistical significance of differences was evaluated by means of 1-way ANOVA; *p < 0.05; **p < 0.01; *N.S*. not significant. (**b**) Mice were fed with HFD with soybean oil for 2 months. Mice were intraperitoneally injected with either 12-HEPE (100 ng/mouse) or vehicle at the last 4 consecutive days, and then pulsatility index and resistive index were examined 1 day after the final injection with 12-HEPE. Data are combined from 2 independent experiments. Each point represents data from individual mice; n = 7–8/group. Statistical significance of differences was evaluated by means of Mann–Whitney test; **p < 0.01.

We next examined whether 12-HEPE acts as an anti-atherosclerosis metabolite. To address this point, we first asked whether the amount of 12-HEPE increases in serum after intraperitoneal injection with 12-HEPE. We found that the amount of 12-HEPE was increased at 60 min after the injection, and declined at the basal level by 120 min (Supplementary Fig. S4). We then intraperitoneally injected 12-HEPE (3 times/week) to mice fed with HFD supplemented with soybean oil. When 12-HEPE was injected during the HFD-feeding, we found that 12-HEPE improved the pulsatility index and resistive index, suggesting that 12-HEPE is effective in prevention of the development of atherosclerosis (Fig. 5a). Moreover, when 12-HEPE was injected to mice that had been maintained on HFD supplemented with soybean oil for 2 months, we found that therapeutic treatment with 12-HEPE injection in this way exerted amelioration of pulsatility index and resistive index (Fig. 5b). These results suggest that 12-HEPE is effective both in preventive and therapeutic manners.

### 12-HEPE inhibited foam cell formation in a PPARγ-dependent manner

**Figure 6:**
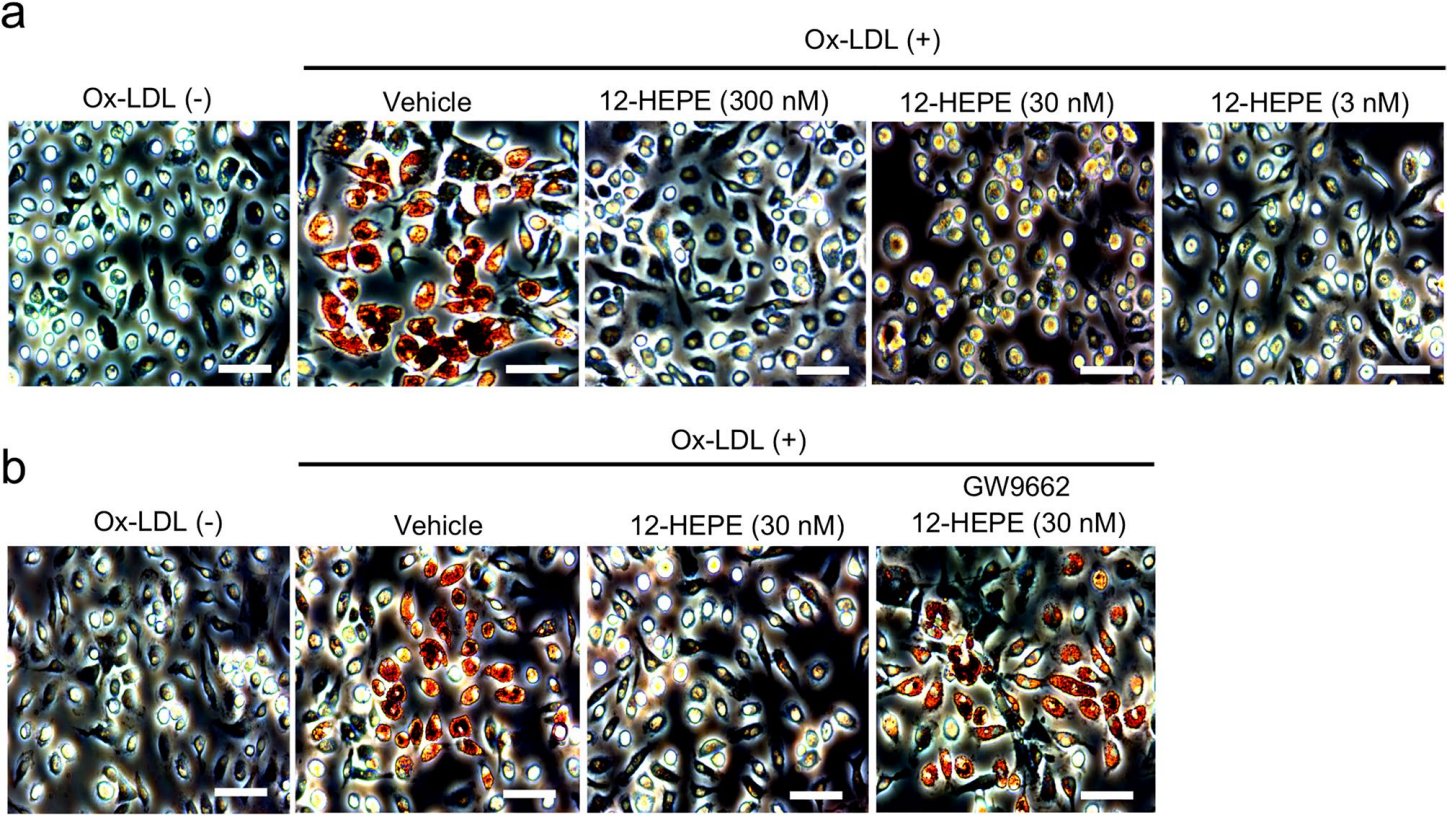
12-HEPE inhibited foam cell formation in a PPARγ-dependent manner. Foam cell formation was induced by stimulating isolated peritoneal cells with Ox-LDL in vitro, and evaluated by staining cells with oil red O solution. (**a**) 12-HEPE was added at the indicated concentrations to the samples at 24 h before stimulation with Ox-LDL. (**b**) 12-HEPE and GW9662 was added to the samples at 24 h and 26 h before stimulation with Ox-LDL, respectively. Data are representative of 2 independent experiments (duplicate assay/experiment). Scale bars 100 μm.

We next investigated the mechanism of action of 12-HEPE in inhibition of the development of atherosclerosis. Because dietary intake of linseed oil led to the inhibition of foam cell formation, we sought to examine whether 12-HEPE directly affected macrophage phenotype. To address this issue, we used in vitro foam cell assay. In this experiment, we prepared peritoneal macrophages and stimulated with Ox-LDL to induce foam cell formation in vitro. When macrophages were stimulated with Ox-LDL, we confirmed that macrophages developed to foam cells (evaluated by oil red O staining; Fig. 6a). Strikingly, foam cell formation was inhibited by exogenous treatment with 12-HEPE (Fig. 6a). We also found that the activity of 12-HEPE was cancelled in the presence of GW9662, a PPARγ antagonist, indicating that 12-HEPE inhibited foam cell formation in a PPARγ-dependent manner (Fig. 6b). Further, the activity of 12-HEPE for anti-foam cell formation is supported by finding that 12-HEPE increased the expression level of *Abca1* and *Abcg1* which act as cholesterol efflux transporters (Supplementary Fig. S5a). We also found that 12-HEPE elevated gene expression levels of some PPARγ-target genes such as *Lxra* and *Arg1* (Supplementary Fig. S5b)^28^, consistent with its action through PPARγ. These results collectively suggest that the 12-HEPE-PPARγ axis acted on macrophages to inhibit Ox-LDL-induced foam cell formation through induction of cholesterol efflux transporters.

## Discussion

In this study, we investigated the impact of dietary ω3 fatty acid and its metabolite 12-HEPE on the development of atherosclerosis. High cholesterol diet and/or *Ldlr*^*−*/−^ mice or *Apoe*^*−*/−^ mice are frequently used in atherosclerosis research area^19,29^; however, some studies evaluated atherosclerosis in C57BL/6 wild-type mice by feeding with HFD although the phenotypes (e.g., LDL level and plaques) were milder than *Ldlr*^*−*/−^ mice or *Apoe*^*−*/−^ mice^30–32^. Consistently, we confirmed that wild-type mice fed with HFD supplemented with soybean oil for 10 months showed milder levels (50–100 mg/dL) of serum LDL/VLDL compared with *Ldlr*^*−*/−^ mice or *Apoe*^*−*/−^ mice (300–500 mg/dL)^32^. In addition, HFD with linseed oil did not decrease the LDL/VLDL level, which is consistent with a previous report showing that PPARγ agonist, rosiglitazone, inhibited the development of atherosclerosis without affecting serum cholesterol level^27^. Process of atherosclerosis lesion development includes (1) vascular activation, (2) monocyte infiltration, (3) macrophage and foam cell development (fatty streak), (4) smooth muscle cell infiltration and extracellular matrix production (fibrous plaque), (5) matrix metalloproteinase production (advanced plaque), and (6) degradation of the extracellular matrix by metalloproteinases (vulnerable plaque and thrombus)^19^. Based on these mechanisms, the current animal model using wild-type mice with HFD for a long period develops up to the state of fatty streak, but not later stages on plaque formations. These findings suggest that evaluating the pulsatility and resistive indexes may catch up proceedings of atherosclerosis prior to the development of large size of lesion plaques.

The anti-inflammatory effect of ω3 fatty acids was first noted in the 1960s, when an epidemiological study by Dyerberg and Bang that compared the diets of the Inuit people of Greenland with those of Danish residents found that daily ω3 fatty acid intake reduces the risk of death from cardiovascular disease^20–22^. Since then, the anti-inflammatory actions of ω3 fatty acids have been extensively studied, and the α-linolenic acid metabolites EPA and DHA have been shown to antagonize the metabolism of the ω6 fatty acid arachidonic acid by the COX and LOX pathways, helping to prevent disease by inhibiting the production of inflammatory eicosanoids^33–35^. Recent technological developments in LC–MS/MS have also enabled the identification of trace compounds in the body, and metabolites with EPA or DHA as their precursors that exhibit potent anti-inflammatory and inflammation-resolving activity; among these, 17,18-EpETE, 15-HEPE and 14-hydroxy-DPA as well as resolvins, protectins, and maresins, have been successively identified^4–7,36^.

Previous epidemiological studies have shown that daily EPA intake inhibits the development of atherosclerosis, but its mechanism of action, including the identification of the active molecule, was hitherto unknown. In this study, we identified the EPA-derived metabolite 12-HEPE as an active molecule that inhibits the transformation of macrophages into foam cells. The fatty acid metabolite 12-HEPE is generated by the action of 12-LOX with EPA as its precursor. 12-LOX is expressed by leukocyte, including macrophages and eosinophils, as well as by platelets and other cells. Progressive atherosclerosis reportedly afflicts 12/15-LOX knockout mice, and 12-LOX activity is believed to play an important role in inhibiting the development of atherosclerosis^37–39^.

Given that macrophages have been observed around the aortic valves of mice fed a diet containing linseed oil, it is conceivable that 12-HEPE is produced as a result of the 12-LOX activity that macrophages themselves possess, and that this activity acts locally via the autocrine and paracrine systems. 12-HEPE reportedly activates PPARγ^40^. A previous study has found that activation of PPARγ induces *Cd36* gene transcription, suggesting the promotive role of PPARγ in the uptake of OX-LDL and may enhance foam cell formation^41^. However, later studies revealed that PPARγ agonists, such as rosiglitazone and quercetin, did not promote foam cell formation, rather it inhibited foam cell formation through induction of ABCA1 and ABCG1 and acceleration of efflux of intracellular cholesterol esters^42–45^. These evidence suggest that 12-HEPE may inhibit macrophage transformation into foam cells by inducing these transporters in macrophages. Indeed, we confirmed that 12-HEPE increased the expression levels of *Abca1* and *Abcg1* in cultured macrophages in vitro.

PPARγ is also known to make macrophages more inclined to become M2 macrophages with anti-inflammatory properties^46–48^, increasing the likelihood that the macrophages in local blood vessels will be of the M2 type. M2 macrophages have been shown to exert an inhibitory effect on the development of atherosclerosis^49–51^, and the inclination of macrophages toward the M2 type as a result of PPARγ activation by 12-HEPE may be one mechanism suppressing the onset of atherosclerosis.

The development of atherosclerosis is intimately connected with the adipocytes surrounding blood vessels^52^, and when adipocytes that have become hypertrophic due to obesity produce the chemokine CCL2, inflammatory M1 macrophages that express its receptor CCR2 migrate to surround them^53^. We previously reported that dietary linseed oil ameliorated HFD-induced elevation of CCL2 and IL-12 in serum^54^, suggesting that linseed oil exerts anti-inflammatory actions to macrophages. Adipocytes also release large amounts of palmitic acid, and stimulation of the Toll-like receptor TLR4 expressed by macrophages causes the production of the inflammatory cytokines TNF-α and IL-6^53^. TNF-α inhibits the production of adiponectin, which is secreted by adipocytes, thus blocking adiponectin’s action in inhibiting inflammatory cytokine production^52^. This leads to the production of even larger amounts of TNF-α and IL-6 by macrophages, resulting in further induction of macrophage invasion and of the expression of Ox-LDL receptors such as CD36 and SR-A, thereby promoting the transformation of macrophages into foam cells^55,56^. Therefore, this suggests that the production of inflammatory chemokines and the secretion of inflammatory cytokines by macrophages, which start with obesity-associated hypertrophy, are closely related to the development of atherosclerosis. PPARγ activation is known to promote adiponectin secretion^57,58^. This means that even if macrophages have aggregated as a result of adipocyte hypertrophy, the secretion of anti-inflammatory cytokines such as adiponectin promoted by the PPARγ-mediated actions by ligands such as 12-HEPE may prevent the subsequent development of atherosclerosis.

In summary, we demonstrated that dietary linseed oil inhibited the development of HFD-induced fatty streak without affecting obesity. 12-HEPE was identified as a dominant ω3 fatty acid metabolite in serum of mice fed with linseed oil, and acted as anti-atherosclerosis molecules by inhibiting foam cell formation in a PPARγ-dependent manner.

## Materials and methods

### Mice

One hundred fifty C57BL/6J wild-type male mice (age; 8 weeks) were purchased from Japan SLC (Hamamatsu, Japan), and maintained for several months on diets composed of chemically defined materials with 4% (wt/wt) and 10% (wt/wt) each dietary oils for control and HFD, respectively (Oriental Yeast, Tokyo, Japan), in the specific pathogen-free animal facility at National Institutes of Biomedical Innovation, Health and Nutrition (NIBIOHN; Osaka, Japan). Detailed diet composition (wt/wt) are as follows: for control diet; casein 14%, l-cystine 0.18%, cornstarch 46.5692%, pregelatinized cornstarch 15.5%, sucrose 10%, cellulose powder 5%, AIN-93M mineral mixture 3.5%, AIN-93 vitamin mixture 1%, choline bitartrate 0.25%, tertiary butylhydroquinone 0.0008% and either soybean oil or linseed oil 4%, for HFD; casein 25%, l-cystine 0.375%, pregelatinized cornstarch 14.869%, sucrose 20%, beef tallow 5%, lard 15%, cellulose powder 5%, AIN-93 M mineral mixture 3.5%, AIN-93 vitamin mixture 1%, choline bitartrate 0.25%, tertiary butylhydroquinone 0.0006% and either soybean oil or linseed oil 10%. In some experiments, mice were preventively treated with 12-HEPE (Cayman chemical; dose 100 ng/mouse) or 0.5% (vol/vol) ethanol in PBS as a vehicle control intraperitoneally 3 times/week during the experimental period. In some experiments, mice were therapeutically treated with 12-HEPE (dose 100 ng/mouse) or 0.5% (vol/vol) ethanol in PBS as a vehicle control at the last 4 consecutive days and pulsatility index was examined 1 day after the final injection. Mice were maintained under conditions (16:8 h light/dark cycle, 22–24 ℃, and 50–60% humidity), with ad libitum access to food and distilled water. When mice were seriously injured by excess fighting during the breeding, we excluded those injured mice from the analysis. All experimental protocols were approved by the Animal Care and Use Committee of NIBIOHN (DS25-2, DS26-41, DS27-47, DSR01-2 and DSR01-3), and all animal experimental procedures were performed according to the Animal Protection Guidelines of NIBIOHN. The study was carried out in compliance with the ARRIVE guidelines.

### Measurement for pulsatility index and resistive index

Pulsatility index was measured by using the Vevo2100 imaging system (FUJIFILM, Toronto, ON, Canada). Blood flow at the murine common carotid artery was visualized by Pulsed-Wave Doppler mode to measure peak-systolic velocity (PSV), endo-diastolic velocity (EDV) and time-averaged maximum flow velocity (TAMV; Vmean). Pulsatility index and resistive index were calculated by (PSV-EDV)/Vmean, and (PSV-EDV)/PSV, respectively^23–25^.

### Histological analysis

Frozen tissue was analyzed histologically as described previously with some modification^2^. In brief, heart samples were washed with PBS (Nacalai Tesque) on ice and frozen in Tissue-TeK OCT compound (Sakura Finetek, Tokyo, Japan) in liquid nitrogen. Frozen tissue sections (6 μm) were prepared by using a cryostat (model CM3050 S; Leica). For oil red O staining, sections were dried by air and washed in water for 30 s, followed by 1 min at room temperature in 60% (vol/vol) 2-propanol (Nacalai Tesque). Sections were then incubated with oil red O solution for 15 min at 37 °C, followed by 1 min at room temperature in 60% (vol/vol) 2-propanol, and 5 min at room temperature in Mayer hematoxylin solution (Wako). Oil red O solution were prepared as follows: 0.3 g oil red O powder (Sigma-Aldrich) was dissolved in 100 ml of 60% (vol/vol) 2-propanol, which was diluted by adding 67 ml of water with stirring for 15 min, and filtered through PES (pore size: 0.22 μm) with Filter System (Corning), and used as oil red O solution. For immunohistologic analysis, frozen tissue sections (6 μm) were fixed for 30 min at 4 °C in prechilled 95% ethanol (Nacalai Tesque) followed by 1 min at room temperature in prechilled 100% acetone (Nacalai Tesque). Sections were washed with PBS for 10 min and then blocked in 2% (vol/vol) newborn calf serum in PBS for 30 min at room temperature in an incubation chamber (Cosmo Bio, Tokyo, Japan). Sections were incubated with primary antibodies in 2% (vol/vol) newborn calf serum in PBS for 16 h at 4 °C in the incubation chamber, washed once for 5 min each in 0.1% (vol/vol) Tween-20 (Nacalai Tesque) in PBS and PBS only, and then stained with secondary antibodies in 2% (vol/vol) newborn calf serum in PBS for 30 min at room temperature in the incubation chamber. To visualize nuclei, sections then were washed twice (5 min each) with PBS and stained with DAPI (AAT Bioquest, Sunnyvale, California, USA; 1 μM) for 10 min at room temperature in the incubation chamber. Finally, sections were washed twice with PBS, mounted in Fluoromount (Diagnostic BioSystems, Pleasanton, California, USA), and examined under a fluorescence microscope (model BZ-9000; Keyence, Osaka, Japan). The following antibodies and reagent were used for immunohistological analysis: purified-anti-F4/80 mAb (Biolegend; 123102; 1:100), Cy3-anti-rat IgG (Jackson Immunoresearch Laboratories; 712-165-153; 1:200), and BODIPY493/503 (Molecular probes; D3922; 1:1000).

### Flow cytometry analysis

Flow cytometry analysis was performed as reported with modifications^59^. Briefly, the upper side of the heart, containing aortic valve was isolated and cut into small pieces by using scissors and incubated for 30 min at 37 °C with stirring in RPMI1640 medium (Sigma-Aldrich) containing 2% (vol/vol) newborn calf serum (Equitech Bio) and 0.5 mg/mL collagenase (Wako Chemicals). Cell suspensions were filtered through cell strainers (70 μm; BD Bioscience), and stained with following mAbs: PE-Cy7 anti-mouse F4/80 (BioLegend; 1:100), APC-Cy7 anti-mouse CD11b (BioLegend; 1:100) and BV421 anti-mouse CD4 (BioLegend; 1:100) to detect macrophages. Anti-CD16/32 mAb (BioLegend; 1:100) and 7-AAD (BioLegend; 1:100) were used to prevent nonspecific staining and detect dead cells, respectively. Samples were analyzed by FACSAria II (BD Bioscience). Data were analyzed by using FlowJo 9.9 (Tree Star).

### Lipidomic analysis

Lipidomic analysis was performed by LC–MS system as reported with modifications^5^. In brief, samples were obtained by using centrifugal columns (Monospin C18-AX, GL Sciences, Tokyo, Japan) after the addition of deuterium-labeled internal standard [15-hydroxyeicosatetraenoic acid (HETE)-d8, arachidonic acid-d8, and leukotriene D_4_-d4; Cayman Chemical, Ann Arbor, MI, USA]. Fatty acid metabolites were analyzed by using a UPLC system (Aquity; Waters, Milford, MA, USA) coupled with a mass spectrometer (Orbitrap Elite; ThermoFisher Scientific, Waltham, MA, USA). UPLC separation was performed with a 1.7-mm, 1.0 × 150 mm Aquity UPLC BEH C18 column (Waters). Mass spectrometric analysis for quantification was based on the ion-trap MS2 detection method in anion mode. Data analysis was performed by using the software Xcalibur 2.2 (ThermoFisher Scientific).

### Foam cell assay

Foam cell assay was conducted in vitro as described previously with modification^60^. Eight-week-old C57BL/6J wild-type mice were intraperitoneally injected with 0.5 mL of 4% (wt/vol) thioglycolate medium (Sigma-Aldrich) and peritoneal cells were collected after 3 days. Peritoneal cells were plated onto 12 well plates (5.0 × 10^5^ cells/ mL /plate; Falcon) in RPMI-1640 (Sigma-Aldrich) containing 10% (vol/vol) FBS and incubated for 2 h at 37 °C in a 5% CO_2_ incubator (Sanyo, Osaka, Japan). After 2 h, non-adherent cells were removed by washing with plain RPMI-1640, and remaining adherent cells were incubated in 1 mL of RPMI-1640 containing 10% (vol/vol) FBS and 50 U/mL penicillin and 50 μg/mL streptomycin (Nacalai Tesque) for 24 h at 37 °C in a 5% CO_2_ incubator. After the incubation, cells were washed with plain RPMI-1640, and incubated with 1 μM GW9662 (Abcam plc, Cambridge, UK) or 0.1% (vol/vol) ethanol as control for 2 h, followed by incubation with 12-HEPE or 0.1% (vol/vol) ethanol as a control for 24 h at 37 °C in a 5% CO_2_ incubator. After the incubation, cells were washed with plain RPMI-1640, and incubated with 50 μg/mL Ox-LDL (Funakoshi) for 48 h at 37 °C in a 5% CO_2_ incubator. After the incubation, cells were washed with D-PBS(−) (Nacalai Tesque), and fixed with 1 mL of 4% PFA (Nacalai Tesque) for 30 min at room temperature, followed by washing with D-PBS(−). Then, samples were incubated in 1 mL of 60% (vol/vol) 2-propanol (Nacalai Tesque) for 1 min at room temperature, followed by incubation in 2 mL of oil red O solutions for 30 min at room temperature. Then, samples were washed with D-PBS, and examined under the microscope (model DM IL LED; Leica).

### ELISA for LDL/VLDL

The amount of LDL/VLDL in the serum was analyzed by using cholesterol assay kit-HDL and LDL/VLDL (Abcam). Absorbance at OD_570_ was measured with the iMark microplate reader (Bio-Rad).

### Reverse transcription and quantitative PCR analysis

Total RNA was isolated and reverse transcription was conducted as described previously^2^. Quantitative PCR analysis was performed with LightCycler 480 II (Roche) with FastStart Essential DNA Probes Master (Roche). Primer sequences were as follows: *Abca1* sense, 5′-atggagcagggaagaccac-3′; *Abca1* antisense, 5′-gtaggccgtgccagaagtt-3′; *Abcg1* sense, 5′-gggtctgaactgccctacct-3′; *Abcg1* antisense, 5′-tactcccctgatgccacttc-3′; *Lxra* sense, 5′-caggaagagatgtccttgtgg-3′; *Lxra* antisense, 5′-tcttccacaactccgttgc-3′; *Arg1* sense, 5′-gaatctgcatgggcaacc-3′; *Arg1* antisense, 5′-gaatcctggtacatctgggaac-3′ ; *Actinb* sense, 5′-aaggccaaccgtgaaaagat-3′; *Actinb* antisense, 5′-gtggtacgaccagaggcatac-3′.

### Statistical analysis

Statistical significance was evaluated through one-way ANOVA for comparison of multiple groups and Mann–Whitney test for two groups (Prism 6, GraphPad Software, La Jolla, CA, USA). A *P* value less than 0.05 was considered to be significant.

## Supplementary Information

Supplementary Information 1.
