# Supplementary material for: 12-Hydroxyeicosapentaenoic acid inhibits foam cell formation and ameliorates high-fat diet-induced pathology of atherosclerosis in mice
Source: Sci Rep. 2021 May 17;11:10426. doi: 10.1038/s41598-021-89707-1 (PMC8129127; doi:10.1038/s41598-021-89707-1)
Supplement: Supplementary file 1 — Supplementary Information 1. [file 41598_2021_89707_MOESM1_ESM.pdf]

Supplementary Information

Title:
12-hydroxyeicosapentaenoic acid inhibits foam cell formation and ameliorates high-fat diet-induced pathology of atherosclerosis in mice

Authors:
Takahiro Nagatake, Yuki Shibata, Sakiko Morimoto, Eri Node, Kento Sawane, So-ichiro Hirata, Jun Adachi, Yuichi Abe, Junko Isoyama, Azusa Saika, Koji Hosomi, Takeshi Tomonaga, and Jun Kunisawa

Figure legends for Supplementary Figures:

Supplementary Fig. S1. Analysis of pulsatility index and resistive index. Mice were fed with HFD with soybean oil or HFD with linseed oil for 4 months (a) and 6 months (b). Pulsatility index and resistive index in common carotid artery were examined. Each point represents data from individual mice; n=8-12/group. Statistical significance of differences was evaluated by means of Mann-Whitney test; ***p<0.001; **p<0.01.

Supplementary Fig. S2. Analysis of serum LDL/VLDL level. Mice were fed with either control diet with soybean oil, HFD with soybean oil or HFD with linseed oil for 10 months, and serum samples were prepared for the analysis of LDL/VLDL. Each point represents data from individual mice; n=4-5/group.

Supplementary Fig. S3. Numbers of macrophages were comparable between mice fed with HFD with soybean oil and linseed oil. Mice maintained on the indicated HFDs for 10 months, and the heart was examined for the counting of macrophages (7-AAD$^-$CD45$^+$F4/80$^+$CD11b$^+$) by flow cytometry. Numbers of macrophages were calculated on the basis of total cell numbers and flow cytometric data. Each point represents data from individual mice; n=6-7/group. Statistical significance of differences was evaluated by means of Mann-Whitney test; N.S., not significant.

Supplementary Fig. S4. Chronological analysis of the amount of 12-HEPE in serum after intraperitoneal injection with 12-HEPE. Mice maintained on a conventional diet

was intraperitoneally injected with 12-HEPE (100 ng/mouse), and the serum was obtained at the indicated time points, and examined the amount of 12-HEPE by LC-MS/MS. Data are expressed as mean ±SEM (n=4-5/group). Statistical significance of differences was evaluated by means of Mann-Whitney test; ***p*<0.01.

Supplementary Fig. S5. 12-HEPE elevated gene expression levels of cholesterol efflux transporters and PPARγ-target genes. Isolated peritoneal cells were stimulated with 12-HEPE at the indicated concentrations or vehicle control (0.5% ethanol/PBS) *in vitro*. 6 hr after the stimulation, total RNA was extracted and cDNA samples were prepared for quantitative RT-PCR analysis for the evaluation of cholesterol efflux transporters (a) and PPARγ-target genes (b). The expression level of each gene was normalized by internal control, *Actinb*, and the data are shown as the ratio to vehicle control. Data are expressed as mean ±SEM (n=4/group). Statistical significance of differences was evaluated by means of 1-way ANOVA; ***p*<0.01

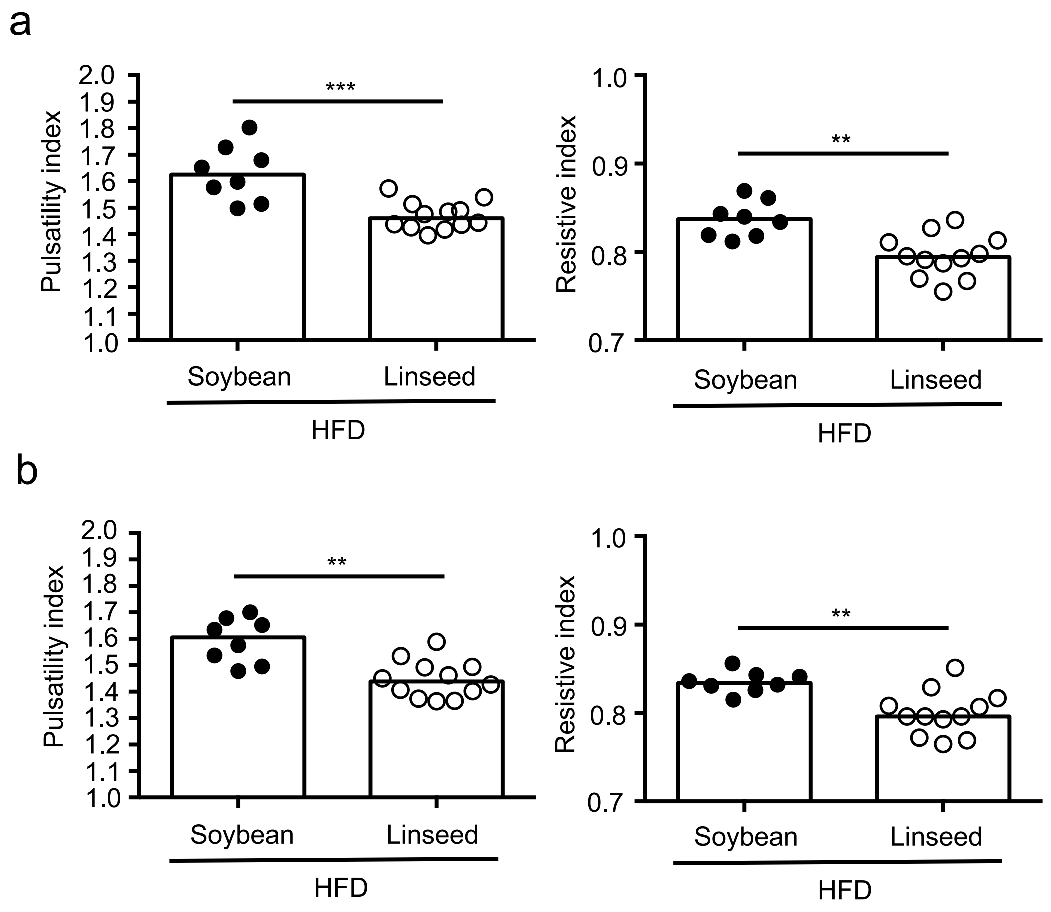

Supplementary Fig. S1

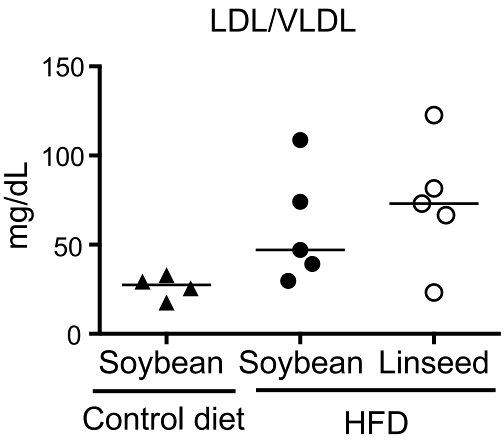

Supplementary Fig. S2

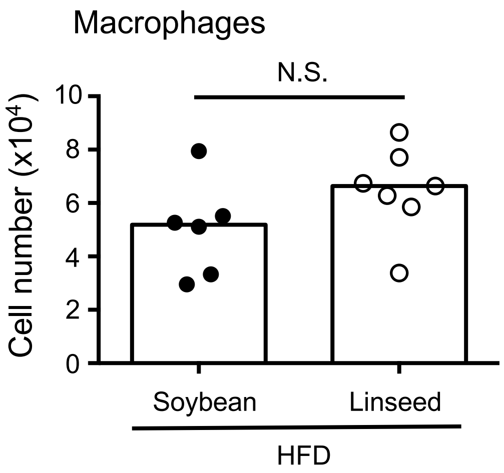

Supplementary Fig. S3

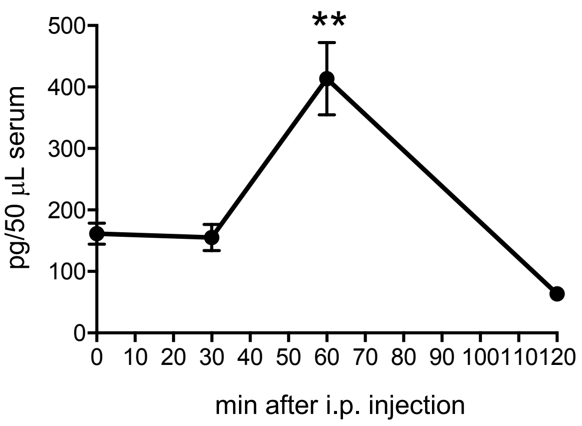

Supplementary Fig. S4

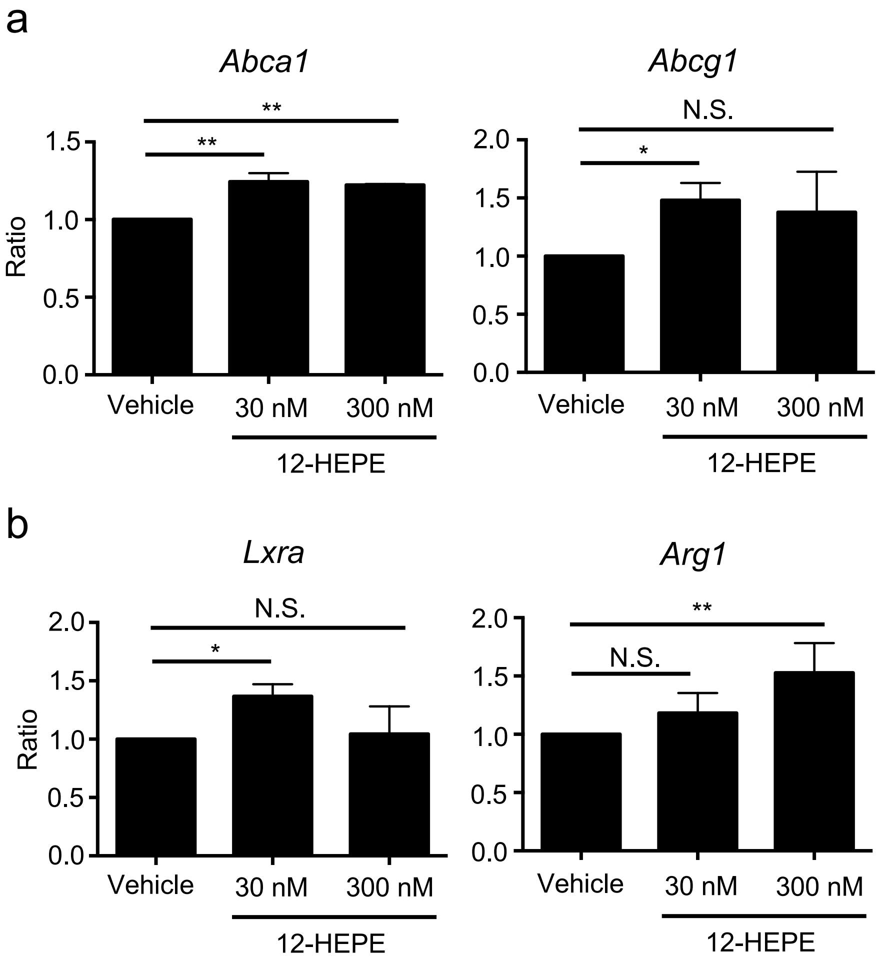

Supplementary Fig. S5
